# Levels of particulate matters in air of the Gonabad city, Iran

**DOI:** 10.1016/j.mex.2018.11.001

**Published:** 2018-11-08

**Authors:** Seyed Ali Sajjadi, Zahra Atarodi, Amir Hossein Lotfi, Ahmad Zarei

**Affiliations:** a Department of Environmental Health Engineering, School of Health, Gonabad University of Medical Sciences, Gonabad, Iran; b Research Committee, Gonabad University of Medical Sciences, Gonabad, Iran

**Keywords:** Air pollution, Particulate matters, Gonabad

## Abstract

The pollution of air and its effect on the ecosystem and particularly on human wellbeing is an issue of important public and governmental concern. To understand the levels of particulate matters and compositional data for total particulate matters fractions, samples were collected from October to February 2018. The samples were collected using gravitational dust sampling method. Weekly and monthly variations of particulate matters in studied stations were in range of 0.0262–0.0802 and 0.0243–0.2109 mg/cm^2^.day. The XRD analysis showed that most of particles were inorganic in nature, representing that these particles originates mainly from deserts. However, local and regional and international sources of these particles should be determined. Thus, comprehensive control efforts will be required in addition to local initiatives to improve the air quality in cities like Gonabad.

## Graphical abstract

**Figure:**
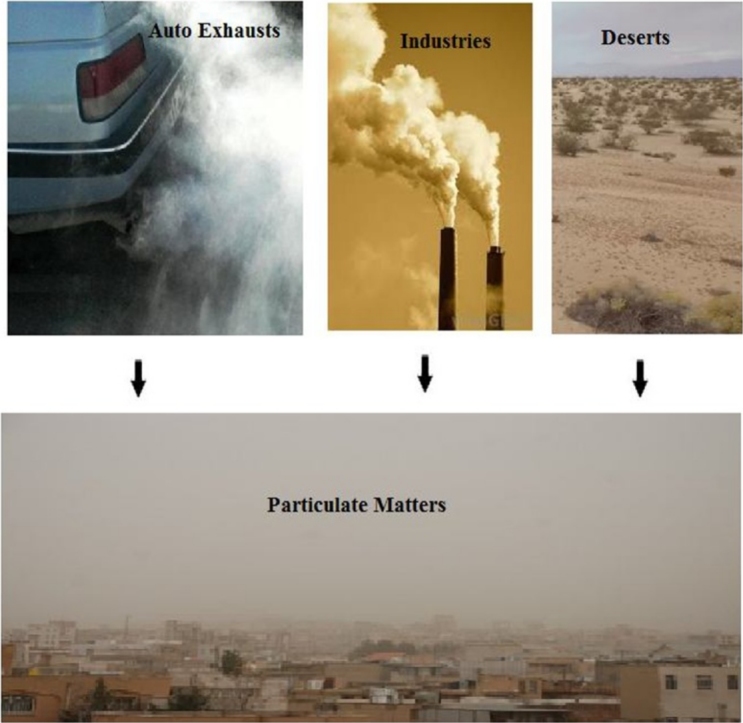

**Specifications Table**

**Table:** 

Subject area	Environmental Science
• More specific subject area:	• Particulate matters in air
• Protocol name:	• Levels of particulate matters in air of the Gonabad city
• Reagents/tools:	• No applicable
• Experimental design:	• Particulate matters were measured using a dust fall instrument.
• Trial registration:	• No applicable
• Ethics:	• No applicable

## Protocol data

• Understanding the levels of particulate matters in air have important public health consequences considering the increasing trend of air pollution of many cities with these pollutants.
• High levels of particulate matters in air can cause a verity of health problems in human based on their composition and properties.
• In this work, total particulate matters were analyzed in 5 public different stations in Gonabad city.

## Description of protocol

Air pollution is considerable environmental health issue as it is reported that around 7 million people died in 2012 as a result of air pollution exposure from both outdoor and indoor emission sources [1]. Particulate matters (PMs) are well-known to have harmful impacts on human health [[2], [3], [4]]. Many studies have indicated a statistical relation between particulate maters and health issues, including respiratory and cardiovascular and genotoxic hazards [[5], [6], [7], [8], [9]]. The particles are also a major cause of visibility impairment (regional haze) in many areas [10] which can affect on transportation as well as tourism. Atmospheric particulate matters are composed of a mixture of solid and aqueous matters which enter the atmosphere by human activities and natural processes [11,12]. Natural sources are dust storms, volcanic eruptions, forest and grassland fires, plants and ocean spray [8]. Anthropogenic resources include traffic, domestic heating, power plants and various industrial processes [13]. Considering the large number of natural and anthropogenic sources, particulate matters may present in diverse physical and chemical characteristics in different regions. Some people are more susceptible from the exposures to particulate matter which includes children, individuals with asthma, and the elderlies with illnesses like bronchitis, emphysema and pneumonia. Patients with chronic obstructive pulmonary disease, such as emphysema and bronchitis, are also potentially susceptible [4,14]. The particles also can affect on building materials, plants etc. The air pollution challenge of particulate maters is especially problematic in many cities of Iran including Ahwaz, Khorramabad, Tehran, Sabzavar, Mashhad, Zabol, Zahedan, Kermanshah, Gonabad etc. where particulate pollution is two to ninety times higher [2,[15], [16], [17], [18], [19]]. Because of these impacts, particulate matter levels are regularly monitored in numerous countries and managed according to the local, regional and in international standards.

### Study area description

**Fig. 1:**
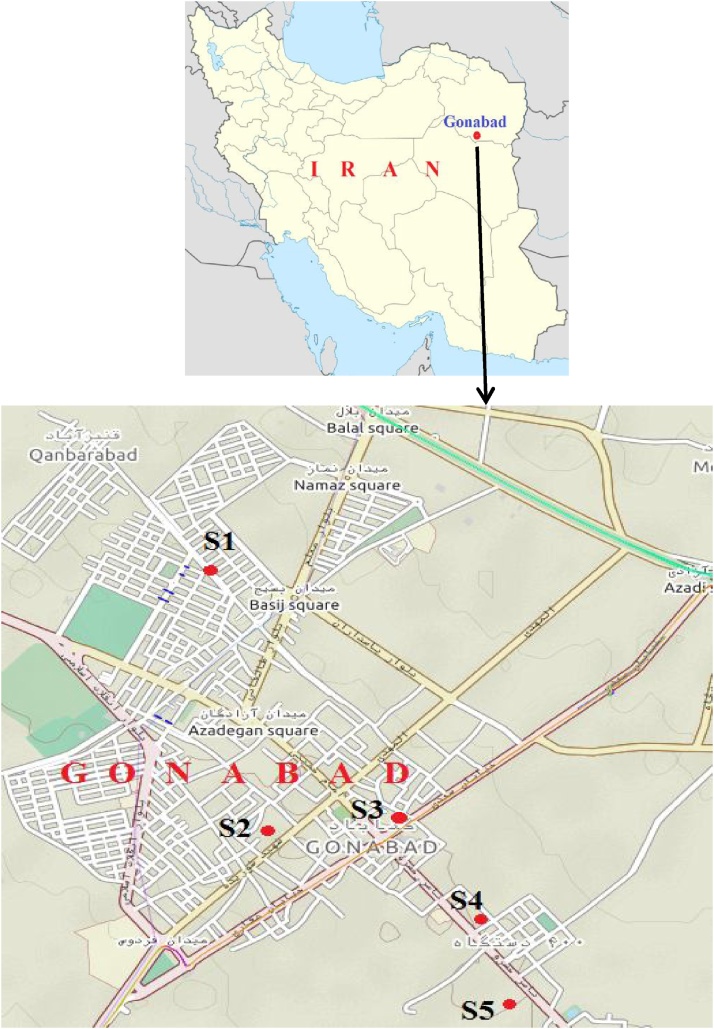
Location of sampling sites in Gonabad.

This study was conducted in Gonabad city. This city is the capital of Gonabad County, in south of Razavi Khorasan Province, western Iran with coordinates 34°21′10″N-58°41′01″E. Its population was 36,367 in 2011, in 10,389 families. Its elevation from sea level is 1150 m. The average annual temperature is 17.3 °C with average precipitation 155 mm. Dust storms occurs several times during year. These storms are due to the regional dust events and also due to the existence of several desert points around Gonabad which act as the source of local dusts. The trend of desertification in south of khorasan province is remarkably increased, especially due to little rainfall and consecutive droughts, overgrazing, and vast destruction of native plants and especially Tamarix trees used for soil stabilization and dust control. The sampling locations in Gonabad are shown in Fig. 1. The sampling period was from October to February 2018.

### Sampling and analysis

In this study, the particulate matter samples were collected using gravitational dust sampling method [20] from October to February 2018. During collection of dust fall samples with the help of petri dish method, the weather was dry. All plastics used for the tests were prewashed with 20% HCl, thoroughly rinsed with deionized water, kept in plastic sacs to avoid contamination before being used. Five suitable locations were selected for placing petri dishes approximately 1000 m to 4000 m apart from each other within the city. The sampling sites were selected about three m above ground level, to prevent any disturbance by animals and the public [20]. Every 24 h, the dishes were collected in the same order in which they were placed. Before sampling, the collection area of each dish was determined. The petri dishes were pre-weighed and post-weighed by a digital scale with an accuracy of 0.0001 g. Finally for determination amount of deposited particles, the difference between initial and final weight of each dish was divided on the surface area of corresponding dish and sampling time. X-ray diffraction pattern was recorded by using the X-ray diffractometer (model XPERT-PRO).

### Levels of particulate matters

**Fig. 2:**
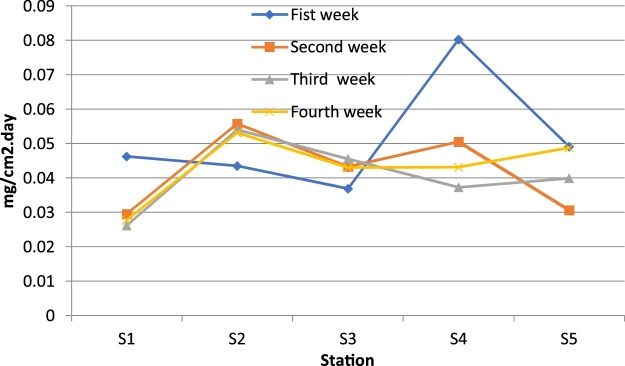
Variations of particulate matters in studied stations in first, second, third and fourth weeks during November.

**Fig. 3:**
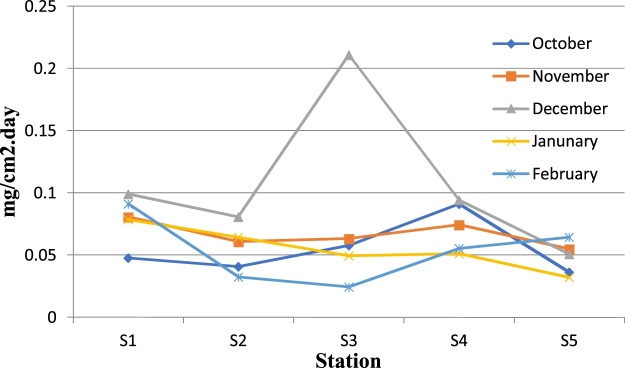
Variations of particulate matters in studied stations in different months.

Variations in the levels of particulate matters are shown in Fig. 2, Fig. 3. and Table 1, Table 2.

**Table 1:** Weekly variations of particulate matters in studied stations in October.

Week	S1	S2	S3	S4	S5
**Fist week**	0.046325	0.043483404	0.03694668	0.0803	0.04916751
**Second week**	0.0295573	0.0558	0.0433	0.0505885	0.0307
**Third week**	0.026200418	0.0540	0.0456	0.0372852	0.03990525
**Fourth week**	0.027812751	0.05321	0.0431	0.04321	0.048732

**Table 2:** Monthly variations of particulate matters in studied stations.

Month	S_1_	S_2_	S_3_	S_4_	S_5_
**October**	0.047576718	0.020838687	0.057720193	0.0913	0.036329768
**November**	0.080638504	0.060818409	0.063449771	0.074569396	0.0550
**December**	0.099185361	0.080596063	0.290935236	0.09434705	0.05089079
**January**	0.07855	0.06423	0.049432	0.05121	0.0321
**February**	0.091016941	0.032321	0.02433	0.05543	0.06433

### XRD analysis

**Fig. 4:**
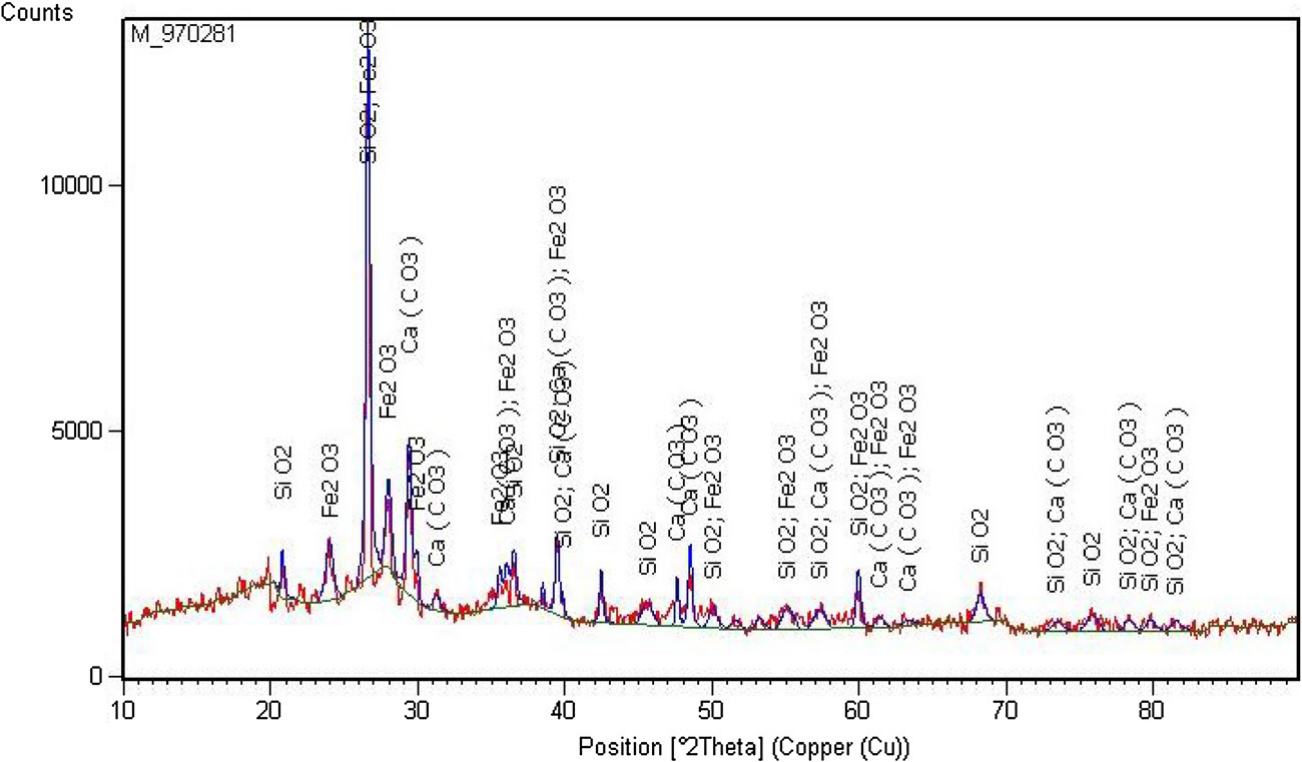
XRD pattern of a representative composite sample of collected particulate matters.

XRD analysis was made to identify the crystallographic structure of the sampled particulate matters (Fig. 4).
